# A Case of Ruptured Uterus, with the Appearances on Dissection

**Published:** 1800

**Authors:** Isaac Cathrall

**Affiliations:** Physician at Philadelphia.


XIV. *A Caſe of ruptured Uterus, with the Appearances on Diſſection. By* Iſaac Cathrall, *M. D. Phyſician at Philadelphia. Communicated in a letter to* John Clarke, *M. D. Phyſician in London; and by him to Dr.* Simmons.

ON the 26th of October 1796, I was ſent for to Mrs. M.— of a robuſt habit of body, and thirty eight years of age, who, after going through the full term of geſtation, had been delivered the preceding morning of a dead child. She

She informed me that it was the firſt ſhe had ever borne, and that during her pregnancy ſhe had experienced, at times, a pain in the right ſide of the abdomen, as low down as the ſpine of the ilium ; and that for ſeveral months previous to parturition ſhe could not lie down on her right ſide ; but notwithſtanding this indiſpoſition, the labour was naturally accompliſhed, though it was lingering, from the head of the child being detained within the pelvis ; and from the recent marks of injury the body of the child exhibited, it in all probability expired during its paſſage through that cavity. After the expulſion of the child, the abdomen appeared augmented in ſize, though the tumor was not circumſcribed, but diffuſed, and very tenſe to the feel, like the abdomen in tympanites. She complained of no other pain than ſtricture acroſs the thorax, with great difficulty in breathing, and a ſenſe of ſuffocation, particularly when attempting to aſſume an horizontal poſture ; this compelled her to be bolſtered up in an arm chair to facilitate reſpiration. Her pulſe was full and ſtrong, attended with an increaſe of heat, conſiderable thirſt, and obſtinately coſtive bowels. On making an examination, *per vaginam*, a quantity of coagulated blood ſucceeded the introduction of the hand; and inſtead of diſcovering another child, as the midwife imagined, I paſſed my hand through an opening in the right ſide of the uterus, into the cavity of the abdomen. The ſource of all the unpromiſing ſymptoms being now aſcertained, I directed ſixteen ounces of blood to be taken from the arm, to palliate the moſt urgent ſymptoms, and to afford the lacerated uterus ſome opportunity of recovering itſelf. To remove the conſtipation of the bowels, one table ſpoonful of caſtor oil was ordered to be taken every half hour, and expedited in its operation by the adminiſtration of laxative clyſters. The next day I found that ſhe had paſſed a ſleepleſs night in a ſitting poſition, and had ſeveral copious ſtools, without any abatement of the tenſion of the abdomen, or difficulty of breathing. Her pulſe now began to fail faſt, with an aggravation of every other ſymptom, together with a copious diſcharge of fluid and coagulated blood from the uterus, which terminated in ſyncope in which ſhe expired about noon.

Several hours after her death, I paſſed a trocar into the left ſide of the abdomen, in the place recommended for the paracenteſis, and diſcharged discharged one pint of a brown colour-ed fœtid fluid, with a quantity of air: this considerably diminished the size of the abdo-men. On examining the contents of that ca-vity, the intestines were found highly inflamed, and very much distended with air; they ad-hered generally to the peritonaeum, which was likewise in a state of inflammation. The uterus had contracted to about the size of a foot ball, and was ruptured on its right side a little above the cervix; the opening exhibited an irregular or ragged appearance, extending a little way above the brim of the pelvis, being in some places two inches and a half in diameter, round which the uterus was considerably thinner than on the opposite side, but firm to the touch, without any appearance of mortification, ex-cepting a slight discolouration of the uterus round the ruptured part.

To record unsuccessful cases is by no means a desirable undertaking, but the particular reasons of my offering this to the public, is, from a wish to exhibit an assemblage of symp-toms which may lead, under similar circum-stances, to a knowledge of a rupture of the uterus, even when the expulsion of the child has been with tolerable facility accomplished;

 and and when we cannot aſcertain a rupture of the uterus by an examination, *per vaginam*. This is ſometimes the caſe, on account of the contraction of the uterus, and particularly when the rupture is in the fundus of that viſcus. Under theſe circumſtances we are obliged to draw our concluſions of the condition of the the uterus, from the ſtate of the ſymptoms; theſe, I have carefully deſcribed, together with the appearances on diſſection, hoping that they may early ſuggeſt to the practitioner their cauſe, ſo that he may ſpeedily apply his remedies, and direct them with better ſucceſs than was experienced in the preceding caſe.

